# P03-012 - A P268S NOD2 mutation in one Blau patient

**DOI:** 10.1186/1546-0096-11-S1-A208

**Published:** 2013-11-08

**Authors:** M Pardeo, E Cortis, C Bracaglia, R Nicolai, F De Benedetti, A Insalaco

**Affiliations:** 1 Division of Rheumatology, Department of Pediatric Medicine, Ospedale Pediatrico Bambino Gesù, Rome, Italy; 2 Pediatric department, Santa Maria della Stella Hospital, Orvieto, Italy

## Introduction

Blau syndrome (BS) is a rare autosomal dominant, autoinflammatory syndrome characterized by the clinical triad of granulomatous, recurrent uveitis, dermatitis and symmetric arthritis. The arthritis is usually a polyarticular exuberant synovitis and tenosynovitis and represents the characteristic phenotypic feature. Uveitis occurs in most patients and commonly evolves to a panuveitis. In the majority of patients, the disease is characterized by early onset, usually before 3-4 years of age. The gene responsible for BS has been identified in the caspase recruitment domain gene NOD2/CARD15, and the most common mutations were found in codon 334 (R334Q and R334W) [1]. NOD2 is a member of a family of intracellular proteins with N-terminal caspase recruitment domains (CARDs). Since the first report of an association of the NOD2 variants with Crohn disease by Hugot et al. [2], extensive studies have been focused on an association of NOD2 with inflammatory bowel disease (IBD), pediatric Blau syndrome, NOD2-associated autoinflammatory disease (NAID) and rheumatic disease [3].

## Case report

We describe a 4-year-old male, the first child of healthy unrelated parents, who presented at 13 months of age, with arthritis of the ankle and of the second proximal inter-phalangeal of the right hand, without the typical puffy appearance. Laboratory test revealed mild increase in inflammatory parametrs (erythrocyte sedimentation rate 35 mm/h, C-reactive protein 0.77 mg/dl) and the presence of antinuclear antibody (titer 1:640, homogeneous pattern). A diagnosis of ANA positive oligoarticular juvenile idiopathic arthritis was made and an infiltration of the ankle joint with TXA was performed, with insufficient response. Persistence of the ankle arthritis led to initiate treatment with methotrexate that was not associated with clear benefit. Four months later the patient developed recurrent episodes of fever and skin rash on limbs and trunk, with spontaneous resolution, and subsequently recurrent episodes of bilateral anterior uveitis. Based on the presence of persistent arthritis, recurrent uveitis, fever and rash, Blau Syndrome was suspected and molecular analysis of NOD2/CARD15 gene was performed. Sequencing analysis demonstrated a heterozygous c.802C>T mutation (P268S /SNP5) in exon 4.

## Discussion

Until now the c.802C>T mutation (P268S /SNP5) in exon 4 of NOD2 had only been reported in association with Crohn's disease, rheumatoid arthritis, spondylarthropathy and ulcerative colitis. This is, to the best of our knowledge, the first case of c.802C>T mutation (P268S /SNP5) that appears to be associated with typical clinical features of Blau syndrome.

## Competing interests

None Declared.
